# Cardiovascular and Renal Effectiveness of GLP-1 Receptor Agonists vs. Other Glucose-Lowering Drugs in Type 2 Diabetes: A Systematic Review and Meta-Analysis of Real-World Studies

**DOI:** 10.3390/metabo12020183

**Published:** 2022-02-15

**Authors:** Irene Caruso, Angelo Cignarelli, Gian Pio Sorice, Annalisa Natalicchio, Sebastio Perrini, Luigi Laviola, Francesco Giorgino

**Affiliations:** Department of Emergency and Organ Transplantation Section of Internal Medicine, Endocrinology, Andrology and Metabolic Diseases, University of Bari Aldo Moro, 70124 Bari, Italy; ireneca91@gmail.com (I.C.); angelo.cignarelli@uniba.it (A.C.); sorice.gianpio@gmail.com (G.P.S.); annalisa.natalicchio@uniba.it (A.N.); sebastio.perrini@uniba.it (S.P.); luigi.laviola@uniba.it (L.L.)

**Keywords:** type 2 diabetes, cardiovascular disease, renal disease, kidney, MACE, real-world evidence, GLP-1 receptor agonists, SGLT-2 inhibitors

## Abstract

Cardiovascular outcome trials (CVOT) showed that treatment with glucagon-like peptide-1 receptor agonists (GLP-1RA) is associated with significant cardiovascular benefits. However, CVOT are scarcely representative of everyday clinical practice, and real-world studies could provide clinicians with more relatable evidence. Here, literature was thoroughly searched to retrieve real-world studies investigating the cardiovascular and renal outcomes of GLP-1RA vs. other glucose-lowering drugs and carry out relevant meta-analyses thereof. Most real-world studies were conducted in populations at low cardiovascular and renal risk. Of note, real-world studies investigating cardio-renal outcomes of GLP-1RA suggested that initiation of GLP-1RA was associated with a greater benefit on composite cardiovascular outcomes, MACE (major adverse cardiovascular events), all-cause mortality, myocardial infarction, stroke, cardiovascular death, peripheral artery disease, and heart failure compared to other glucose-lowering drugs with the exception of sodium-glucose transporter-2 inhibitors (SGLT-2i). Initiation of SGLT-2i and GLP-1RA yielded similar effects on composite cardiovascular outcomes, MACE, stroke, and myocardial infarction. Conversely, GLP-1RA were less effective on heart failure prevention compared to SGLT-2i. Finally, the few real-world studies addressing renal outcomes suggested a significant benefit of GLP-1RA on estimated glomerular filtration rate (eGFR) reduction and hard renal outcomes vs. active comparators except SGLT-2i. Further real-world evidence is needed to clarify the role of GLP-1RA in cardio-renal protection among available glucose-lowering drugs.

## 1. Introduction

Since 2008, a plethora of cardiovascular outcomes trials (CVOT) have been conducted to assess the cardiovascular and renal safety of new glucose-lowering drugs, with unexpectedly beneficial evidence for glucagon-like peptide-1 (GLP-1) receptor agonists (GLP-1RA) and sodium-glucose transporter-2 (SGLT-2) inhibitors (SGLT-2i) on cardio-renal outcomes [1]. Large randomized controlled trials, like the CVOT, allow to avoid some of the confounding factors typical of everyday clinical practice by imposing strict selection criteria, random allocation of treatments, standard data collection, and sustained adherence [2]. However, the strict requirements of randomized controlled trials come at the expense of generalizability, as their results are reliably informative only for populations with the same features and in a similar setting of care [2]. For instance, Sciannameo et al. demonstrated that only 45.4% of patients with type 2 diabetes from a large Italian database could be eligible for at least one of the GLP-1RA CVOT [3]. Moreover, eligible patients were still different from actual trial participants for several relevant demographic and anthropometric characteristics and comorbidity profiles (e.g., older, poorer glycemic control, lower prevalence of cardiovascular complications, higher prevalence of microvascular complications) [3].

The scientific community has been paying increasing attention to real-world studies, using data from electronic medical records, registries, health insurance claims, or surveys, which could provide clinicians with evidence closer to their everyday clinical practice in broader populations or usually underrepresented subgroups in a less controlled environment [1,2]. Nonetheless, the interpretation of the results of real-world studies should take into account their several limitations, such as the confounding, selection, information, and reporting biases, only some of which are avoidable with the refined statistical strategies implemented in recent studies [4]. The aim of this review is to summarize the available evidence from real-world studies on cardiovascular and renal effectiveness of GLP-1RA, placing it in the context of the available literature. First, data from observational studies concerning the effects of GLP-1RA on atherosclerosis and cardiovascular risk factors will be discussed; then, the comparisons between GLP-1RA and other glucose-lowering drugs and between GLP-1RA and SGLT-2i will be addressed, focusing on their effect on composite cardiovascular outcomes and single endpoints, such as stroke, myocardial infarction, cardiovascular death, peripheral artery disease, and heart failure. Finally, the exiguous real-world studies concerning kidney function, mainly investigating changes in eGFR and albuminuria rather than hard renal endpoints, will be reviewed.

## 2. Materials and Methods

**Figure 1:**
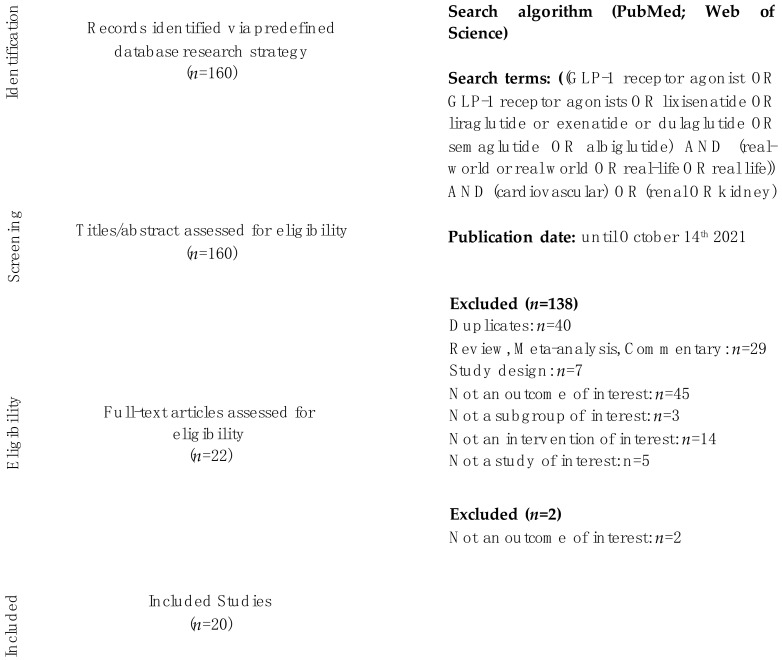
PRISMA flowchart: identification of eligible trials.

The aim of this study was to summarize the effect of GLP-1RA vs. other glucose-lowering drugs on main cardiovascular (MACE, myocardial infarction, stroke, cardiovascular death) and renal outcomes in dedicated real-world patients with type 2 diabetes. A systematic literature search was conducted from 1st August to 14th October 2021, in PubMed and Web of Science, using the following terms: “GLP-1RA”, “GLP-1 receptor agonists”, “lixisenatide”, “liraglutide”, “semaglutide”, “exenatide”, “dulaglutide”, “real-world”, “real-life”, “cardiovascular”, “renal”, “kidney”, “stroke”, “myocardial infarction”, “all-cause death”, “mortality”. References of primary articles were also taken into consideration. Search excluded conference abstracts. Conflicts over study inclusion were settled by consensus. Trial eligibility was independently confirmed by Francesco Giorgino. One hundred and sixty papers were identified. As a result of the screening process, a total of twenty trials was identified. Data extraction was independently performed by Irene Caruso and Angelo Cignarelli. The meta-analysis was restricted to cardiovascular outcomes, due to the heterogeneity and paucity of the results of real-world studies with renal outcomes. The paper by Nørgaard et al. was not included in the meta-analysis as we were not able to retrieve the hazard ratio for the outcomes of interest. The meta-analysis was conducted by Irene Caruso and Angelo Cignarelli with Review Manager (RevMan) version 5.4, The Cochrane Collaboration, 2020. This systematic review was conducted in accordance with the PRISMA reporting guideline (Figure 1). This study is registered with PROSPERO, number CRD42021288260.

## 3. Results

### 3.1. Glucagon-like Peptide-1 Receptor Agonists (GLP-1RA) and Cardiovascular Outcomes in Real-World Studies

#### 3.1.1. Atherosclerosis and Cardiovascular Risk Factors

A meta-analysis of 40 randomized controlled trials with a median follow-up of 6 months showed that treatment with GLP-1RA was associated with significant improvement of biomarkers of inflammation and oxidative stress, such as C-reactive protein, TNF-alpha, malondialdehyde, and adiponectin in overweight type 2 diabetic patients with a mean HbA1c of 55.6 mmol/mol (7.26%), compared to placebo or conventional glucose-lowering drugs [5]. Specifically, experimental studies suggested that liraglutide was able to hinder the progression of atherosclerosis and improve plaque stability [6]. Furthermore, in small placebo-controlled randomized controlled trials, the combination of metformin and liraglutide may reduce the levels of the most atherogenic LDL subfraction and C-reactive protein in patients with coronary artery disease and new onset type 2 diabetes [7]. Compared to sitagliptin, liraglutide was also found to increase VEGF, which is a mediator of angiogenesis and might attenuate the adverse consequences of large vessel atherosclerosis by inducing the formation of collateral vessels in obese type 2 diabetic patients [8]. As recently summarized by Ussher et al., GLP-1RA-mediated cardiovascular protection could be mainly attributed to their vascular anti-inflammatory and anti-atherosclerotic actions, alongside improvements in heart glucose metabolism, fibrosis, oxidative stress, cardiomyocytes viability, and, even though to a small extent, in blood pressure [9].

Real-world studies add to this scenario by confirming GLP-1RA-mediated anti-atherosclerotic effects in routine clinical practice. The prospective observational study conducted by Rizzo et al. was among the first to show that adding liraglutide 1.2 mg/day to metformin was able to improve cardio-metabolic risk factors in 121 individuals with type 2 diabetes and metabolic syndrome after 18 months [10]. The authors also reported a significant reduction in carotid intima-media thickness, a surrogate marker of early atherosclerosis, suggesting a correlation with liraglutide-mediated improvement in lipid profile [10]. Nikolic et al. further investigated this association, demonstrating that this treatment regimen led to lower carotid intima-media thickness through reduction in atherogenic small dense LDL concentrations in 62 type 2 diabetic patients followed for 4 months [11]. These studies reinforce the extraglycemic relevance of GLP-1RA, which might exert significant benefits on early stage atherosclerosis, preventing plaque formation and progression, as suggested by mechanistic studies [11].

Beside direct anti-atherosclerotic effects, real-world studies also showed that GLP-1RA have a beneficial impact on cardiovascular risk factors and, consequently, cardiovascular risk scores. In a multicenter retrospective observational study, Frison et al. followed 103 patients for 5 years and demonstrated that treatment with liraglutide yielded a significant reduction in the 5- and 10-years risk of fatal and non-fatal coronary heart disease based on the UKPDS (United Kingdom Prospective Diabetes Study) score, and this was associated with a sustained benefit on HbA1c, systolic and diastolic blood pressure, and total cholesterol levels [12]. Similarly, a small study conducted on 105 type 2 diabetic patients showed that adding liraglutide to metformin, but not to sulphonylureas, for at least 48 months was linked to a significant reduction in patients’ cardiovascular risk measured with the Framingham risk score [13]. Furthermore, in small cohort studies conducted in type 2 diabetic patients, liraglutide and exenatide exhibited benefits in line with those seen in RCT on glycemic control, body weight, blood pressure, total cholesterol and triglycerides levels, and other known cardiovascular risk factors over a follow-up period of 36 and 6 months, respectively [14,15].

#### 3.1.2. Cardiovascular Outcomes in Real-World Studies Comparing Patients Initiating Glucagon-like Peptide-1 Receptor Agonists (GLP-1RA) vs. Conventional Glucose-Lowering Drugs

CVOT showed that GLP-1RA administration reduced the risk of major adverse cardiovascular events (MACE), defined as non-fatal myocardial infarction, non-fatal stroke and cardiovascular death, by 14% (HR 0.86, 95% CI 0.80–0.93, I^2^ = 44.5%) compared to placebo in type 2 diabetic patients, most of whom with ascertained cardiovascular disease [16]. This overall benefit reflected a significant reduction in myocardial infarction (HR 0.90, 95% CI 0.83–0.98, I^2^ = 26.9%), cardiovascular death (HR 0.87, 95% CI 0.80–0.94, I^2^ = 13.4%) and, even more strikingly, stroke (HR 0.83, 95% CI 0.76–0.92, I^2^ = 0%) [16]. GLP-1RA were also shown to lower the risk of secondary endpoints such as all-cause mortality (HR 0.88, 95% CI 0.82–0.94, I^2^ = 10.1%) and hospitalization for heart failure (HR 0.89, 95% CI 0.82–0.98, I^2^ = 3.0%) [16].

**Figure 2:**
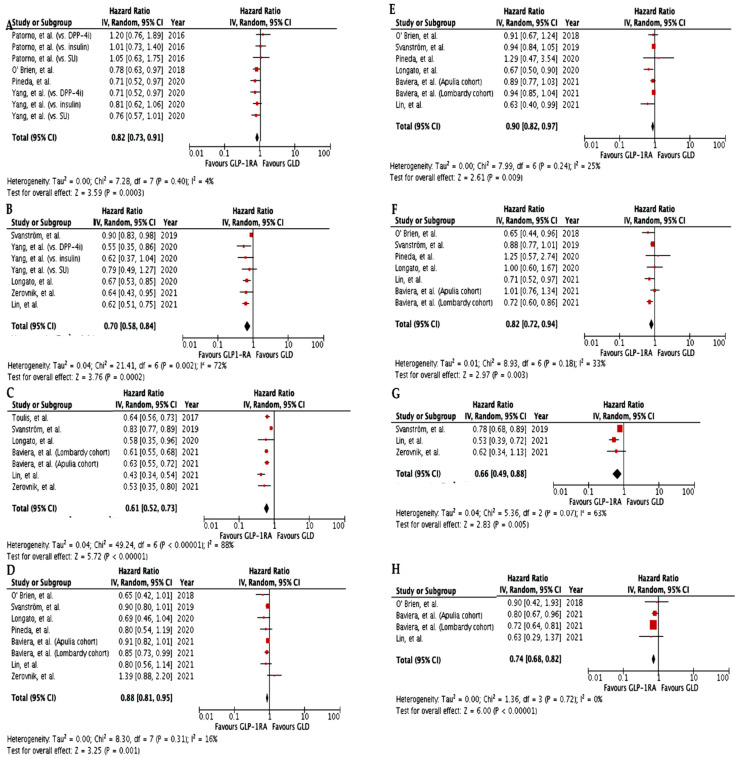
Meta-analysis of the CV effects in RWS comparing GLP-1RA vs. other GLD (except SGLT-2i). (**A**). Effect of GLP-1RA vs. other GLD on CV composite outcome; (**B**). Effect of GLP-1RA vs. other GLD on MACE (CV death, non-fatal MI, or non-fatal stroke); (**C**). Effect of GLP-1RA vs. other GLD on all-cause death; (**D**). Effect of GLP-1RA vs. other GLD on HF; (**E**). Effect of GLP-1RA vs. other GLD on MI; (**F**). Effect of GLP-1RA vs. other GLD on stroke; (**G**). Effect of GLP-1RA vs. other GLD on CV death; (**H**). Effect of GLP-1RA vs. other GLD on PAD. GLP-1RA, glucagon-like peptide-1 receptor agonists; GLD, glucose-lowering drugs; SGLT-2i, sodium-glucose transporter-2 inhibitors; CV, cardiovascular; MACE, major adverse cardiovascular events; HF, heart failure; MI, myocardial infarction; PAD, peripheral arterial disease.

Real-world studies investigating cardiovascular outcomes differ from CVOT, allowing comparison between GLP-1RA and other glucose-lowering drugs instead of placebo, in the absence of clinical trial procedures, in populations with more heterogeneous characteristics (e.g., ethnicity, gender, age, prevalence of macrovascular disease) and in the setting of everyday clinical practice. Compared to other glucose-lowering drugs, with the exception of SGLT-2i, treatment with GLP-1RA yielded favorable results (Table 1, Figure 2). Four studies investigated a composite cardiovascular outcome as the primary endpoint, which was, however, different in each study. Pineda et al. found that GLP-1RA initiation significantly reduced the risk of the composite cardiovascular outcome by 29% [17], while Yang et al. and O’Brien et al. demonstrated a 27% [18] and 22% [19] risk reduction vs. DPP-4i users, respectively; in contrast, Patorno et al. observed no significant difference between treatment groups [20]. GLP-1RA produced a significant reduction in MACE risk, ranging from 10 to 45% when compared to DPP-4i [18,21,22,23,24]. We carried out a meta-analysis of the cardiovascular effects of GLP-1RA vs. other glucose-lowering drugs as observed in real-world studies. Overall, GLP-1RA significantly reduced the composite cardiovascular outcome by 18% (HR 0.82, 95% CI 0.73–0.91, I^2^ = 4%) and MACE by 30% (HR 0.70, 95% CI 0.58–0.84, I^2^ = 72%) (Figure 2A,B). All-cause mortality was found to be also reduced to an even greater extent with GLP-1RA use in all considered studies regardless of the comparator [21,22,23,24,25,26] (HR 0.61, 95% CI 0.52–0.73, I^2^ = 88%) (Figure 2C). Only some studies investigated cardiovascular death, suggesting a significant benefit on this particular outcome (HR 0.66, 95% CI 0.49–0.88, I^2^ = 63%) (Figure 2G). The evidence on myocardial infarction and stroke mirrors the results in the CVOT. Even though Longato et al. and Lin et al. found that GLP-1RA administration was associated with a lower risk of myocardial infarction only when compared to DPP-4i [21,23], our meta-analysis of RWS showed a significant 10% myocardial infarction risk reduction with a low degree of heterogeneity (HR 0.90, 95% CI 0.82–0.97, I^2^ = 25%) (Figure 2E). In line with the remarkable benefit on stroke observed in CVOT vs. placebo, real-world studies demonstrated that GLP-1RA initiation was associated with an 18% reduction in the risk of stroke vs. other glucose-lowering drugs (HR 0.82, 95% CI 0.72–0.94, I^2^ = 33%) (Figure 2F); however, only Baviera et al., Lin et al., and O’Brien et al. showed a significant GLP-1RA-mediated cerebrovascular protection [19,23,25]. Baviera et al., using data from administrative health databases of two highly populated Italian regions, Lombardy and Apulia, found significant cerebrovascular protection only in the Lombardy cohort (HR 0.70, 95% CI 0.63–0.79 vs. HR 1.02, 95% CI 0.86–1.21) [25]. Differences in population size (approximately double in Lombardy), baseline cerebrovascular risk (higher in Apulia), and glucose control during follow-up might account for this discrepancy; however, pooling data from the two regions failed to confirm the cerebrovascular protection observed in the Lombardy cohort [25]. This study was also the only one showing a significant benefit in both cohorts in the risk of hospitalization for peripheral artery disease and lower limb complications (HR 0.72, 95% CI 0.64–0.82/ HR 0.67, 95% CI 0.56–0.81, and HR 0.80, 95% CI 0.67–0.98/HR 0.69, 95% CI 0.51–0.93, respectively) in GLP-1RA users with respect to those on other glucose-lowering drugs [25]. Our meta-analysis of the three real-world studies assessing peripheral artery disease occurrence showed a significant 25% risk reduction of this particular outcome (HR 0.74, 95% CI 0.68–0.82, I^2^ = 0%) (Figure 2H).

In spite of their heavy impact on patients’ wellbeing, major adverse limb events have been scarcely studied in randomized controlled trials with GLP-1RA [23]. A post-hoc analysis of the LEADER trial suggested a beneficial role of liraglutide compared to placebo on preventing amputations in patients with diabetic foot ulcers [27]. The large retrospective Taiwanese cohort study by Lin et al. focused on the effect of GLP-1RA on a composite outcome comprising incident peripheral artery disease, critical limb ischemia, lower limb angioplasty or by-pass, and amputation compared to dipeptidyl peptidase-4 inhibitors (DPP-4i) [23]. Over a mean follow-up period of 2.2 years, the risk of major adverse limb events was significantly lower in new users of GLP-1RA (HR 0.63, 95% CI 0.41–0.96), and this was mainly driven by a lower risk of amputations [23]. Interestingly, a subgroup analysis found that GLP-1RA-mediated protection from major adverse limb events was higher in patients without established cardiovascular disease, hinting at their preferential effects in the early stages of atherosclerosis [23].

The meta-analysis of five studies addressing the risk of heart failure-related outcomes as a secondary endpoint [17,19,21,22,25] showed an overall 12% reduction in initiators of GLP-1RA vs. other glucose-lowering drugs (HR 0.88, 95% CI 0.81–0.95, I^2^ = 16%) (Figure 2D). However, in none of the individual meta-analyzed studies, was there a significant difference between GLP-1RA and other glucose-lowering drugs new users [17,19,21,22,23,24,25]. Accordingly, another previously published meta-analysis of ten observational studies reporting heart failure-related outcomes in type 2 diabetic patients treated with GLP-1RA found conflicting results: in three cohort studies GLP-1RA use was associated with heart failure prevention, in three cohort and three nested case-control studies no differences with comparators emerged, while in one study GLP-1RA use was inferior vs. comparator [28]. The use of active comparators (i.e., DPP-4i, insulin, sulphonylureas), the short duration of type 2 diabetes and consequently the low risk of heart failure, the small number of events accrued, and the different baseline features of enrolled populations could potentially explain why results from observational studies were less prominent compared to CVOT [28]. However, further evidence is required to clarify the role of GLP-1RA in the prevention of heart failure-related outcomes.

#### 3.1.3. Cardiovascular Outcomes in Real-World Studies Comparing Patients Initiating Glucagon-like Peptide-1 Receptor Agonists (GLP-1RA) vs. Sodium-Glucose Transporter-2 Inhibitors (SGLT-2i)

Current guidelines recommend indifferently prescribing GLP-1RA or SGLT-2i to type 2 diabetic patients at high cardiovascular risk or with ascertained atherosclerotic cardiovascular disease, regardless of baseline HbA1c levels [29]. Instead, SGLT-2i should be preferred in patients with heart failure or chronic kidney disease and albuminuria [29]. There are no available head-to-head randomized controlled trials comparing the effect of GLP-1RA and SGLT-2i on cardiovascular outcomes; hence, despite their limitations, real-world studies may be useful to fill this gap.

**Figure 3:**
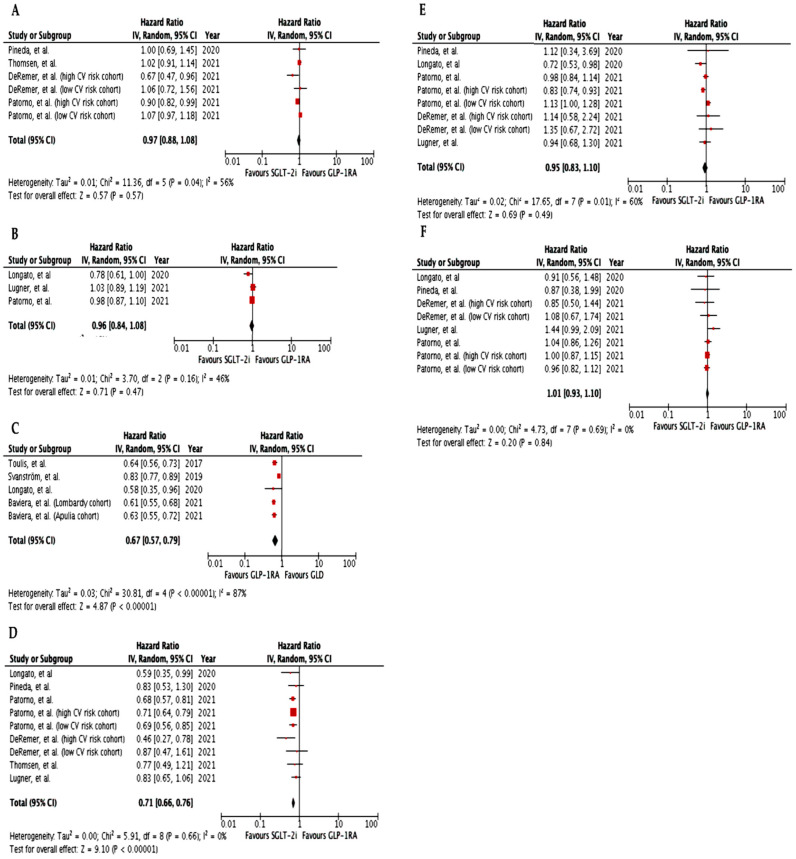
Meta-analysis of the CV effects in RWS comparing GLP-1RA vs. SGLT-2i. (**A**). Effect of GLP-1RA vs. SGLT-2i on CV composite outcome; (**B**). Effect of GLP-1RA vs. SGLT-2i on MACE (CV death, non-fatal MI, or non-fatal stroke); (**C**). Effect of GLP-1RA vs. SGLT-2i on all-cause death; (**D**). Effect of GLP-1RA vs. SGLT-2i on HF; (**E**). Effect of GLP-1RA vs. SGLT-2i on MI; (**F**). Effect of GLP-1RA vs. SGLT-2i on stroke. GLP-1RA, glucagon-like peptide-1 receptor agonists; GLD, glucose-lowering drugs; SGLT-2i, sodium-glucose transporter 2 inhibitors; CV, cardiovascular; MACE, major adverse cardiovascular events; HF, heart failure; MI, myocardial infarction.

Overall, our meta-analysis found no significant difference in the risk of composite cardiovascular outcome (HR 0.97, 95% CI 0.88–1.08, I^2^ = 56%) or MACE (HR 0.96, 95% CI 0.84–1.08, I^2^ = 46%) in type 2 diabetic patients treated with GLP-1RA vs. SGLT-2i (Figure 3A,B). Indeed, most real-world studies showed a similar effect of GLP-1RA vs. SGLT-2i on composite cardiovascular outcomes in patients at low cardiovascular risk [17,30,31,32,33,34,35], with the exception of the study by Longato et al., in which a 22% reduction in the incidence of MACE over a median follow-up of 13 months in SGLT-2i vs. GLP-1RA users was noted (Table 2) [36]. The Authors found that the SGLT-2i benefit was particularly clear in patients with ascertained cardiovascular disease [36]. Accordingly, DeRemer et al. and Patorno et al. showed that SGLT-2i initiators in secondary prevention had a significantly lower risk of composite cardiovascular outcomes vs. GLP-1RA initiators [30,31]. Furthermore, SGLT-2i initiation was associated with greater protection from all-cause death (HR 0.67, 95% CI 0.57–0.79, I^2^ = 87%) vs. GLP-1RA; a subgroup analysis of included studies showed that this benefit was particularly clear in patients with ascertained cardiovascular disease [31,35].

In agreement with randomized controlled trials results, real-world studies confirmed that SGLT-2i were significantly more effective than GLP-1RA in the prevention of hospitalization for heart failure (HR 0.71, 95% CI 0.66–0.76, I^2^ = 0%) (Figure 3D), both in patients with previous cardiovascular disease [30,31] and at low cardiovascular risk [31,33,35,36]. Despite lacking the statistical power to reliably assess the singular components of the composite cardiovascular outcome, SGLT-2i, and GLP-1RA initiators were at a similar risk of myocardial infarction (HR 0.95, 95% CI 0.83–1.10, I^2^ = 60%) and stroke (HR 1.01, 95% CI 0.93–1.10, I^2^ = 0%) (Figure 3E,F). Instead, treatment with SGLT-2i was associated with a higher risk of peripheral artery disease (HR 1.68, 95% CI 1.04–2.72, *p* = 0.035) [32] and lower limb amputations (HR 1.44, 95% CI 1.06–1.96) [35] compared to GLP-1RA. Accordingly, data from Swedish and Danish nationwide registries demonstrated that GLP-1RA users exhibited a significantly reduced risk of lower limb amputations compared to SGLT-2i [37].

### 3.2. Glucagon-like Peptide-1 Receptor Agonists (GLP-1RA) and Renal Outcomes in Real-World Studies

Experimental evidence demonstrated that GLP-1RA exert direct beneficial effects on kidney cells mainly by activating the cAMP/PKA signaling pathway, which is involved in the pathogenesis of increased albuminuria, regulation of proximal tubule sodium reabsorption through NHE3, and modulation of oxidative stress through NADPH, as well as by mitigating the renin-angiotensin-aldosterone system [6]. Furthermore, improvement in traditional risk factors for diabetic kidney diseases, such as hypertension and hyperglycemia, might contribute to lower kidney disease occurrence and progression [6].

Most GLP-1RA CVOT, with the exception of ELIXA, Harmony Outcomes, and PIONEER 6, investigated the effect of GLP-1RA on slightly different composite kidney endpoints, comprising new-onset macroalbuminuria, doubling of serum creatinine or at least 40% reduction in estimated glomerular filtration rate (eGFR), kidney replacement therapy, and death due to kidney disease; in ELIXA, the analysis of kidney-related outcomes was restricted to new-onset macroalbuminuria [16]. A recent meta-analysis of these trials showed a significant reduction in the composite kidney endpoint in patients treated with GLP-1RA compared to placebo (HR 0.79, 95% CI 0.73–0.87) [16], yet these results were mainly driven by prevention of macroalbuminuria, whereas data on hard kidney endpoints were inconclusive due to the low number of events accrued [38]. Moreover, as for cardiovascular outcomes, the generalizability of the renal benefits of GLP-1RA to patients at low cardiovascular risk is largely unknown, even though results from the exploratory analysis of the composite renal outcome in the REWIND trial, enrolling a relevant percentage of the population without overt cardiovascular disease (68.5%), were encouraging [39].

By including a broader population and allowing the accrual of a higher number of hard renal events [4], real-world studies yielded promising results on GLP-1RA-mediated renal protection, which complement those derived from the randomized controlled trials (Table 3). A small observational retrospective study conducted in Italy on 261 patients with a mean eGFR of 83.9 mL/min/1.73 m^2^ and albuminuria of 83.7 mg/L showed that treatment with liraglutide was associated with a non-significant reduction in microalbuminuria by −16.6 mg/L and preserved eGFR at 36 months [40]. Two real-world studies collecting data from the same US electronic health record database investigated to effect of GLP-1RA on eGFR compared to other glucose-lowering drugs in the first year after treatment initiation [41,42]. In 2966 matched pairs of type 2 diabetic patients with preserved kidney function (mean eGFR of 82 mL/min/1.73 m^2^), new users of GLP-1RA exhibited a significantly smaller reduction in eGFR (−0.80 vs. −1.03 mL/min/1.73 m^2^) and were less likely to have a ≥30% reduction in eGFR (2.19 vs. 3.14%) with respect to other glucose-lowering drugs initiators [42]. Similarly, in 1183 matched pairs of type 2 diabetic patients with preserved kidney function (mean eGFR of 83.7 mL/min/1.73 m^2^), initiation of dulaglutide was associated with a smaller decline in eGFR (−0.4 vs. −0.9 mL/min/1.73 m^2^) and a lower risk of ≥30% reduction in eGFR (3.3 vs. 4.1%) compared to insulin glargine [41]. In a population with preserved renal function (mean eGFR >90 mL/min/1.73 m^2^ and micro- and macroalbuminuria in approximately 20% and 4% of patients), a Swedish nationwide observational study showed no significant difference between GLP-1RA and SGLT2i initiation in the incidence rate of either the renal composite outcome of micro- or macroalbuminuria, eGFR 50% reduction or lower than 60 mL/min/1.73 m^2^, dialysis, renal transplantation, renal failure, or renal death, or its individual components; yet, point estimates for most of the kidney outcomes were in favor of SGLT-2i initiators [32]. Interestingly, Pasternak et al. conducted a Scandinavian cohort study on 38,731 matched pairs of new users of GLP-1RA or DPP-4i to investigate hard renal outcomes (hospitalization for renal events, renal replacement therapy, or death from renal causes); the majority of GLP-1RA initiators were on liraglutide (92.5%) and exenatide (6.2%) [38]. Using an intention-to-treat exposure definition, GLP-1RA therapy was associated with a significantly lower risk of the composite renal endpoint (HR 0.76, 95% CI 0.68–0.85) and of its components, hospitalization for renal events (HR 0.73, 95% CI 0.65–0.83) and renal replacement therapy (HR 0.73, 95% CI 0.62–0.87) [38]. This benefit is likely affected by an on-treatment effect, as using an as treated exposure definition, the GLP-1RA-mediated renal protection was found to be even greater (HR 0.60, 95% CI 0.49–0.74).

## 4. Discussion

The GLP-1RA- and SGLT-2i-mediated cardiovascular and renal protection that emerged from CVOT has recently led to a paradigm shift in diabetes care, urging clinicians to implement these new glucose-lowering drugs in patients at high cardio-renal risk, irrespective of glucose control [43]. These beneficial effects were demonstrated with respect to placebo in the strict context of randomized controlled trials. A growing number of real-world studies is underway, aiming to overcome the limits of randomized controlled trials and addressing clinically relevant issues, such as proving cardiovascular benefit in broader and diverse populations and comparing GLP-1RA and other glucose-lowering drugs, especially SGLT-2i, since current guidelines describe them as equally effective in type 2 diabetic patients at high risk or with established atherosclerotic cardiovascular disease [29]. Indeed, compared to CVOT, the results of real-world studies are interesting, as they enrolled populations at lower cardiovascular risk and allowed a broader representation of women and different ethnicities. Notably, Patorno et al. enrolled individuals with a mean age of 72 years, approximately ten years older than those enrolled in the CVOT and also most real-world studies, providing information on an often neglected subgroup of type 2 diabetic patients [35].

However, most real-world studies are limited by the lack of information regarding diabetes duration [17,19,20,22,31] and HbA1c levels [17,18,19,25,31], which are relevant contributors to cardiovascular risk [22], as well as other anthropometric and metabolic features. Lugner et al. reported similar effects of SGLT-2i and GLP-1RA both on metabolic endpoints and cardiovascular outcomes [32]; conversely, Longato et al. found a greater improvement in HbA1c in GLP-1RA vs. SGLT-2i initiators (−0.5% vs −0.4%, *p* = 0.001), while changes in systolic blood pressure and lipid profile significantly favored SGLT-2i users [36]. Mediation and meta-regression analyses of CVOT suggested that HbA1c reduction was a relevant mediator of the GLP-1RA beneficial effect on MACE, and this association seemed to be driven by that between HbA1c lowering and stroke [44]. Hence, the unavailability of data regarding baseline levels and changes in cardiovascular risk factors represent a limitation of most real-world studies investigating cardiovascular outcomes that might have hindered the interpretation of results. Another limitation is represented by the relatively short follow-up duration in most real-world studies exploring the effects of GLP-1RA and SGLT-2i. Indeed, while SGLT-2i seemingly exert cardiovascular protection very early on after initial exposure [45], GLP-1RA likely require several months, especially in populations at low baseline cardiovascular risk [36]; thus, real-world studies might have been too short to detect the actual extent of GLP-1RA-mediated cardiovascular benefit, particularly when compared to SGLT-2i. Furthermore, real-world studies did not include patients on semaglutide [46], efpeglenatide [47], and ertugliflozin [48], which have been associated with significant cardiovascular protection and neutral cardiovascular effects, respectively, in recent dedicated CVOT.

Finally, our meta-analysis is limited by the fact that real-world studies often differ in the definition of the primary composite cardiovascular outcome, active comparators, and use of specific GLP-1RA molecules. Indeed, Patorno et al. failed to show any superiority of GLP-1RA over DPP-4i, sulphonylureas, and insulin, but the majority of GLP-1RA initiators were on exenatide bis in die [20]. Evidence from an administrative database of Veneto, a region in the North-East of Italy, showed that patients treated with human-based GLP-1RA were at lower risk of MACE (HR 0.61, 95% CI 0.39–0.95), myocardial infarction(HR 0.51, 95% CI 0.28–0.94), and hospitalization for cardiovascular causes (HR 0.66, 95% CI 0.28–0.94) compared to those on exendin-based GLP-1RA during a median follow-up of 18 months, regardless of baseline established cardiovascular disease [49]. Of note, exposure time to GLP-1RA is critical to cardiovascular protection, and it could be influenced by pharmacokinetic properties (e.g., lixisenatide has a significantly shorter half-life compared to others GLP-1RA, thus engaging the GLP-1 receptor for a limited period of time), as well as practical matters hampering adherence (e.g., the unhandy device used for exenatide once weekly injection was likely the cause of the lowest exposure to the investigational product observed in the EXSCEL trial compared to other GLP-1RA) [50]. Indeed, excluding lixisenatide initiators, human-based GLP-1RA were still associated with a lower risk of MACE and hospitalization due to cardiovascular causes [49]; however, differences in patients’ adherence rates between groups were not addressed in this study [49] nor in other real-world studies investigating cardiovascular outcomes. While in randomized controlled trials patients’ adherence is actively supported, in everyday clinical practice approximately half of type 2 diabetic patients fail to reach their glycemic target, often due to lack of adherence to glucose-lowering drugs [51]. Carls et al. suggested that poor adherence could account for up to 75% of the gap that can be found between randomized controlled trials and real-world studies results [52]. In real-world studies with GLP-1RA, adherence rates are widely variable but unlikely to be persistently greater than 50% [53]. Dosing frequency (e.g., twice daily, once daily, once weekly) and the features of the delivery device seem to have a prominent impact on adherence rates [53]. The underrepresentation of once-weekly GLP-1RA, with small percentages of dulaglutide users in few studies and semaglutide and easier-to-use exenatide BCise not included due to their recent launch in the market, could have had a relevant impact on real-world studies results.

Real-world studies investigating renal outcomes are exiguous yet encouraging. Specifically, a large Scandinavian cohort study allowed to detect a significant benefit of GLP-1RA on hard renal outcomes, such as renal-replacement therapy, hospitalization, and death due to renal causes, when compared to DPP-4i [38]. Smaller studies suggested a similar risk of eGFR reduction compared to SGLT-2i new users and a significantly higher likelihood of eGFR preservation in GLP-1RA new users with respect to other glucose-lowering drugs.

## 5. Conclusions

Despite its limitations, this analysis of RWS investigating cardio-renal outcomes of GLP-1RA suggested that initiation of GLP-1RA was associated with a greater benefit on composite CV outcomes, MACE, all-cause mortality, myocardial infarction, stroke, CV death, PAD, and HF compared to other GLD, with the exception of SGLT-2i. Pasternak et al. found that GLP-1RA reduced the risk of hard renal outcomes compared to DPP-4i. Initiation of SGLT-2i and GLP-1RA yielded similar effects on composite CV outcomes, MACE, stroke, and MI, whereas the former was more effective in the prevention of HF and all-cause mortality.

### Future Perspectives

Choosing between GLP-1RA or SGLT-2i might soon become obsolete, due to the mounting evidence supporting their additive beneficial CV effect [54]. Yet, reimbursement and costs will likely still be an issue in some countries. RWS allowed the interesting comparison between GLP-1RA and SGLT-2i, as ad hoc RCT are too costly, aiding clinical decision making. Furthermore, future RWS should explore neglected outcomes like PAD and hard renal adverse events, being able to assess large numbers of patients for a considerable amount of time. Moreover, observational studies in populations with established CV disease might detect whether these drugs act differently according to the affected CV district. Overall, further real-world evidence is awaited, possibly with a longer duration of follow-up, and including recently launched compounds and data on patients’ metabolic features, to better understand the role of GLP-1RA in cardio-renal protection among available GLD.

## Figures and Tables

**Table 1:** Summary of CV outcomes in RWS comparing patients initiating GLP-1RA vs. other GLD (SGLT-2i excluded).

Study	N	FU (yrs)	bCVD (%)	Comparators	GLP-1RA	CV Composite Endpoint *	MACE	CV Death	All-Cause Death	Stroke	ACS/MI	PAD	HHF
Baviera, et al., 2021	18,716 ^a^; 9772 ^b^	3.9 ^a^; 3.7 ^b^	11.8–12.1 ^a^ 11.9–12.9 ^b^	MET, SU, glinides, TZD, acarbose, DPP-4i	-	-	-	-	**0.61 (0.56–0.65)** ^a^ **0.63 (0.55–0.71)** ^b^	**0.72 (0.60–0.87) ^a^ 1.01 (0.76–1.33)** ^b^	0.94 (0.85–1.04) ^a^ 0.89 (0.77–1.03) ^b^	**0.72 (0.64–0.82) ^a^ 0.80 (0.67–0.98)** ^b^	0.91 (0.82–1.01) ^a^ 0.85 (0.73–1.00) ^b^
Zerovnic, et al., 2021	855 vs. 3817	2.5–3.2	6	DPP-4i	44.9%Lira, 32.6%Dula, 17.5%Exe, 4.9%Lixi	-	**0.64 (0.43–0.97)**	0.62 (0.34–1.14)	**0.53 (0.35–0.79)**	-	-	-	1.39 (0.88–2.21)
Lin, et al., 2021	4460 vs. 13,380	2.3–3.2	20.2	DPP-4i	-	-	**0.62 (0.51–0.76)**	**0.53 (0.39–0.73)**	**0.43 (0.34–0.54)**	**0.71 (0.52–0.96)**	**0.63 (0.40–0.97)**	0.63 (0.29–1.35)	0.80 (0.56–1.13)
Pineda, et al., 2020	815	1	14.4–23.1	MET, SU, glinides, TZD, acarbose, DPP-4i, insulin	-	**0.71 (0.52–0.90)**	-	-	-	1.25 (0.57–2.73)	1.29 (0.47–3.57)	-	0.80 (0.54–1.17)
Yang, et al., 2020	1893 ^c^, 1829 ^d^, 1367 ^e^	1.5–2	14.9–14.7 ^c^ 14.7–17.0 ^d^ 16.5–18.1 ^e^	DPP-4i ^c^, SU ^d^, insulin ^e^	-	**0.73 (0.57–0.96)** ^c^ **0.76 (0.57–1.00)** ^d^ **0.81 (0.62–1.07)** ^e^	**0.55 (0.35–0.86)** ^c^ **0.79 (0.49–1.26)** ^d^ **0.62 (0.37–1.02)** ^e^	-	-	-	-	-	-
Longato, et al., 2020	2807	1.5	13.7–15.1	DPP-4i	Exe, Lira, Lixi, Dula	-	**0.67 (0.53–0.86)**	-	**0.58 (0.35–0.96)**	1.00 (0.60–1.68)	**0.67 (0.50–0.91)**	-	0.69 (0.46–1.04)
Svanström, et al., 2019	23,402	3.3	81.0	DPP-4i	Lira	-	**0.90 (0.83–0.98)**	**0.78 (0.68–0.91)**	**0.83 (0.77–0.90)**	0.88 (0.77–1.01)	0.94 (0.84–1.06)	-	0.90 (0.80–1.03)
O’Brien, et al., 2018	11,351 vs. 28,898	1.3	5.5	DPP-4i	-	**0.78 (0.63–0.96)**	-	-	-	**0.65 (0.44–0.97)**	0.91 (0.67–1.24)	0.90 (0.42–1.95)	0.65 (0.42–1.02)
Toulis, et al., 2017	8345 vs. 16,541	2.6	21.8–20.4	Conventional GLD	55%Lira, 42%Exe, 3%Lixi	-	-	-	**0.64 (0.56–0.74)**	-	-	-	-
Patorno, et al., 2016	35,534 ^c^, 28,138 ^d^, 47,068 ^e^	0.5–0.8	~20	DPP-4i ^c^, SU ^d^, insulin ^e^	67.1%ExeBID, 28.3%Lira, 4.6%ExeQW	1.20 (0.76–1.89) ^c^ 1.05 (0.63–1.74) ^d^ 1.01 (0.73–1.41) ^e^	-	-	-	-	-	-	-

N, number of pairs or number of patients treated with GLP-1RA vs. comparators; FU, follow-up; bCVD, baseline cardiovascular disease (in GLP-1RA vs. comparator cohorts); CV, cardiovascular; MACE, major adverse cardiovascular events; ACS, acute coronary syndrome; MI, myocardial infarction; PAD, peripheral arterial disease; HHF, hospitalization for heart failure; MET, metformin, SU, sulphonylureas; TZD, thiazolidinediones; DPP-4i, dipeptidyl peptidase-4 inhibitors; SGLT-2i, sodium-glucose transporter-2 inhibitors; GLP-1RA, glucagon-like peptide-1 receptor agonists; Exe, exenatide; Lira, liraglutide; Lixi, lixisenatide; Dula, dulaglutide; BID, bis in die; QW, once weekly; GLD, glucose-lowering drugs. Follow-up is reported as mean or median. Statistically significant results are in bold (*p* < 0.05). ^a^ Lombardy cohort, ^b^ Apulia cohort; ^c^ vs. DPP-4i, ^d^ vs. SU, ^e^ vs. insulin. * The CV composite endpoint varied between studies. Pineda et al.: MI, stroke, unstable angina, or coronary revascularization; Yang et al.: MI, ischemic heart disease, HF, stroke, cardiogenic shock, sudden cardiac arrest, arteriosclerotic CV disease, or arrhythmia; O’Brien et al.: hospitalization for congestive HF, stroke, ischemic heart disease, or peripheral artery disease; Patorno et al., 2016: hospitalizations for acute MI, unstable angina, stroke, or coronary revascularization.

**Table 2:** Summary of CV outcomes in RWS comparing patients initiating GLP-1RA vs. SGLT-2i.

Study	N	FU (yrs)	bCVD(%)	Comparators	GLP-1RA	CV Composite Endpoint *	MACE	CV Death	All-Cause Death	Stroke	ACS/MI	PAD	HF
^§^ Patorno, et al., 2021	45,047	0.5	45.2	SGLT-2i (76.9%Cana, 13.1%Dapa, 11.1%Empa)	58.7% Lira, 23.5% Exe, 14.8% Dula, 3.0% Albi	-	0.98 (0.87–1.10)	0.83 (0.64–1.07)	0.95 (0.81–1.11)	1.04 (0.86–1.27)	0.98 (0.84–1.16)	-	0.68 (0.57–0.80)
^§^ DeRemer, et al., 2021	4829 vs. 7082	n.a.	Subgroup 1: 0 Subgroup 2: 100	SGLT-2i (Empa, Cana)	Exe, Lira, Albi, Dula	Subgroup 1: 1.06 (0.72–1.49) Subgroup 2: 0.67 (0.47–0.96)	-	-	-	Subgroup 1: 1.08 (0.67–1.75) Subgroup 2: 0.85 (0.50–1.70)	Subgroup 1: 1.35 (0.67–2.71) Subgroup 2: 1.14 (0.58–2.15)	-	Subgroup 1: 0.87 (0.47–1.61) Subgroup 2: 0.46 (0.27–0.79)
^#^ Nørgaard, et al., 2021	8913 vs. 5275	n.a.	n.a.	SGLT-2i	-	-	5.6% (5.2–6.1) vs. 5.6% (4.8–6.3)	-	-	2.5% (2.2–2.9) 2.6% (2.2–3.1)	2.1% (1.8–2.4) vs. 2.1% (1.8–2.4)	-	1.7% (1.5–2.0) vs. 1.8% (1.2–2.5)
^§^ Patorno, et al., 2021	Cohort 1: 133,139 Cohort 2: 52,901	0.6	Cohort 1: 0 Cohort 2: 100	SGLT-2i (Cana, Dapa, Empa)	Albi, Dula, Exe, Lira	Cohort 1: 1.07 (0.97–1.18) Cohort 2: 0.90 (0.82–0.98)	-	-	Cohort 1: 1.01 (0.87–1.17) Cohort 2: 0.88 (0.79–0.99)	Cohort 1: 0.96 (0.82–1.13) Cohort 2: 1.00 (0.87–1.15)	Cohort 1: 1.13 (1.00–1.28) Cohort 2: 0.83 (0.74–0.93)	-	Cohort 1: 0.69 (0.56–0.85) Cohort 2: 0.71 (0.64–0.79)
^§^ Thomsen, et al., 2021	12,706 vs. 14,498	1.1	30	SGLT-2i (Empa)	Lira	1.02 (0.91–1.14)	-	-	0.93 (0.89–0.98)	-	-	-	0.77 (0.49–1.20)
^§^ Lugner, et al., 2021	9648 vs. 12,097	1.7–1.1	15.8–17.0	SGLT-2i (56.6%Empa, 43.2% Dapa, 0.2% Cana)	75.1% Lira, 16.3% Dula, 6.4% ExeQW	-	1.03 (0.89–1.21)	1.00 (0.47–2.13)	0.78 (0.61–1.01)	1.44 (0.99–2.08)	0.94 (0.68–1.3)	1.68 (1.04–2.72)	0.83 (0.65–1.07)
^§^ Longato, et al., 2020	8596	1.08	18	SGLT-2i (50% Empa, 40% Dapa, 10% Cana)	48% Dula, 34% Lira, 14% Exe, 4% Lixi	-	0.78 (0.61–0.99)	-	0.74 (0.43–1.29)	0.91 (0.56–1.48)	0.72 (0.53–0.98)	-	0.59 (0.35–0.99)
^§^ Pineda, et al., 2020	947	1	12.8–12.0	SGLT-2i	-	1.00 (0.69–1.44)	-	-	-	0.87 (0.38–1.97)	1.12 (0.34–3.68)	-	0.83 (0.53–1.30)

N, number of pairs or number of patients treated with GLP-1RA vs. comparators; FU, follow-up; bCVD, baseline cardiovascular disease (in GLP-1RA vs. comparator cohorts); CV, cardiovascular; MACE, major adverse cardiovascular events; ACS, acute coronary syndrome; MI, myocardial infarction; PAD, peripheral arterial disease; HHF, hospitalization for heart failure; MET, metformin, SU, sulphonylureas; TZD, thiazolidinediones; DPP-4i, dipeptidyl peptidase-4 inhibitors; SGLT-2i, sodium-glucose transporter-2 inhibitors; Cana, canagliflozin; Dapa, dapagliflozin; Empa, empagliflozin; GLP-1RA, glucagon-like peptide-1 receptor agonists; Exe, exenatide; Lira, liraglutide; Lixi, lixisenatide; Dula, dulaglutide; Albi, albiglutide; BID, bis in die; QW, once weekly; GLD, glucose lowering drugs; n.a., not available. GLP-1RA vs. SGLT2i statistically significant results are in bold; SGLT-2i vs. GLP-1RA statistically significant results are underscored (*p* < 0.05). Follow-up is reported as mean or median. * The CV composite endpoint varied between studies. Pineda et al.: MI, stroke, unstable angina, or coronary revascularization; DeRemer et al.: stroke, MI, or HF; Patorno et al., 2021: hospitalization for ischemic or hemorrhagic stroke or MI; Thomsen et al.: stroke, MI, unstable angina, coronary revascularization, HHF, or all-cause death. ^§^ Results are presented as SGLT-2i vs. GLP-1RA as in the source manuscript. ^#^ Results are expressed as % of risk (95% CI) in GLP-1RA vs. SGLT-2i users as in the source manuscript.

**Table 3:** Summary of renal outcomes in RWS with GLP-1RA vs. other GLD (including SGLT-2i).

		Boye et al.	Boye et al.	Lugner et al.	Pasternak et al.
**Main Baseline Features**	N	5932	2366	21,781	77,462
	GLP-1RA	-	Dulaglutide	75.1% Liraglutide 16.3% Dulaglutide 6.4% Exenatide QW	92.5% Liraglutide 6.2% Exenatide 0.7% Lixisenatide 0.6% Dulaglutide
	Comparator	Other GLD	Insulin Glargine	SGLT-2i (56.6% Empagliflozin 43.2% Dapagliflozin 0.2% Canagliflozin)	DPP-4i
	Follow-up (yrs)	1	1	1.7–1.1	3.0
	Mean age (yrs)	59.2	59.7	60.5	59.3
	Female (%)	52.0	51.1	37.5	40.7
	Mean diabetes duration (yrs)	-	-	7.5	-
	Mean HbA1c (%)	8.4	8.3	8.3	-
	Mean eGFR (mL/min/1.73 m^2^)	82.1	83.7	91.6	-
	eGFR <60 mL/min/1.73 m^2^ (%)	19.4	18.2	-	4.6
	Mean albuminuria (mg/L)	-	-	-	-
	Microalbuminuria (%)	-	-	20.6	-
	Macroalbuminuria (%)	-	-	4.3	-
**Renal Outcomes**	Change in albuminuria (mean (95% CI))	-	-	-	-
	New onset MA (HR (95% CI))	-	-	0.89 (0.77–1.04)	-
	Change in eGFR (mL/min/1.73 m^2^)	**−0.80 vs. −1.03** ***p* = 0.0005**	**−0.4 vs. −0.9** ***p* = 0.0024**	-	-
	≥30% eGFR reduction	**2.19% vs. 3.14%** ***p* < 0.0001**	**3.3% vs 4.1%;** ***p* < 0.0001**	0.92 (0.68–1.25) ^§^	-
	≥40% eGFR reduction (HR (95% CI)) ^#^	-	-	0.94 (0.62–1.43)	-
	Composite renal Outcome * (HR (95% CI)) ^#^	-	-	0.98 (0.92–1.05)	**0.76 (0.68–0.85)/0.60 (0.49–0.74)**
	Renal replacement therapy (HR (95% CI)) ^#^	-	-	-	**0.73 (0.62–0.87)/0.42 (0.29–0.62)**
	Hospitalization for renal events (HR (95% CI)) ^#^	-	-	-	**0.73 (0.65–0.83)/0.63 (0.50–0.78)**
	Renal death (HR (95% CI)) ^#^	-	-	-	**0.72 (0.48–1.10)/**0.66 (0.24–1.49)

GLP-1RA, glucagon-like peptide-1 receptor agonists; SGLT-2i, sodium-glucose cotransporter-2; GLD, glucose lowering drugs; DPP-4i, dipeptidyl peptidase-4 inhibitors; MA, macroalbuminuria. Statistically significant results are in bold. ^§^ HR (95% CI). ^#^ Results are presented as intention-to-treat/as-treated analyses. * Lugner et al.: any of micro- or macroalbuminuria, eGFR 50% reduction or lower than 60, dialysis, renal transplantation, renal failure, renal death; Pasternak et al.: renal replacement therapy, hospitalization for renal causes and death for renal causes.

## Data Availability

Data is contained within the article or supplementary material. The data presented in this study are available in Table 1, Table 2 and Table 3 and summarized in Figure 1 and Figure 2.

## Abbreviations

ACS: acute coronary syndrome; Albi, albiglutide; bCVD, baseline cardiovascular disease; BID, bis in die; cAMP, Cyclic Adenosine Monophosphate; Cana, canagliflozin; CI, confidence interval; CV, cardiovascular; CVOT, cardiovascular outcome trials; Dapa, dapagliflozin; DPP-4i, dipeptidyl peptidase-4 inhibitors; Dula, dulaglutide; eGFR, estimated glomerular filtration rate; Empa, empagliflozin; Exe, exenatide; GLD, glucose-lowering drugs; GLP-1RA, glucagon-like peptide-1 receptor agonists; HF, heart failure; HHF, hospitalization for heart failure; HR, hazard ratio; Lira, liraglutide; Lixi, lixisenatide; LDL, low-density lipoprotein; MACE, major adverse cardiovascular events; MALE, major adverse limb events; MET, metformin; MI, myocardial infarction; NADPH, nicotinamide adenine dinucleotide phosphate; NHE3, sodium–hydrogen exchanger 3; PAD, peripheral arterial disease; PKA, protein kinase A; QW, once weekly; RWS, real-world studies; SGLT-2i, sodium-glucose transporter-2 inhibitors; SU, sulphonylureas; TNF-alpha, tumor necrosis factor alpha; TZD, thiazolidinediones; UKPDS, United Kingdom Prospective Diabetes Study; VEGF, vascular endothelial growth factor.
